# First biphotochromic fluorescent protein moxSAASoti stabilized for oxidizing environment

**DOI:** 10.1038/s41598-022-11249-x

**Published:** 2022-05-12

**Authors:** N. K. Marynich, M. G. Khrenova, A. V. Gavshina, I. D. Solovyev, A. P. Savitsky

**Affiliations:** 1 A.N. Bach Institute of Biochemistry, Research Center of Biotechnology of the Russian Academy of Sciences, Moscow, Russia; 2 Department of Chemistry, Lomonosov Moscow State University, Moscow, Russia

**Keywords:** Biological fluorescence, Protein design, Protein structure predictions, Reaction kinetics and dynamics, Optical spectroscopy

## Abstract

Biphotochromic proteins simultaneously possess reversible photoswitching (on-to-off) and irreversible photoconversion (green-to-red). High photochemical reactivity of cysteine residues is one of the reasons for the development of “mox”-monomeric and oxidation resistant proteins. Based on site-saturated simultaneous two-point C105 and C117 mutagenesis, we chose C21N/C71G/C105G/C117T/C175A as the moxSAASoti variant. Since its on-to-off photoswitching rate is higher, off-to-on recovery is more complete and photoconversion rates are higher than those of mSAASoti. We analyzed the conformational behavior of the F177 side chain by classical MD simulations. The conformational flexibility of the F177 side chain is mainly responsible for the off-to-on conversion rate changes and can be further utilized as a measure of the conversion rate. Point mutations in mSAASoti mainly affect the pK_a_ values of the red form and off-to-on switching. We demonstrate that the microscopic measure of the observed pK_a_ value is the C–O bond length in the phenyl fragment of the neutral chromophore. According to molecular dynamics simulations with QM/MM potentials, larger C–O bond lengths are found for proteins with larger pK_a_. This feature can be utilized for prediction of the pK_a_ values of red fluorescent proteins.

## Introduction

Since the decoding of the GFP gene, fluorescent proteins have become reliable and effective genetically encoded biological markers^1^. To date, a huge color palette of fluorescent proteins has been developed, covering the entire visible spectrum^2,3^. Phototransformations of proteins have also been discovered: photoactivation^4,5^, reversible photoswitching^6^, and irreversible photoconversion^7^. Biphotochromic proteins simultaneously possessing reversible photoswitching and irreversible photoconversion were genetically engineered^8–10^ and discovered in nature^11^. In recent years, work has been actively carried out to increase the chemical inertness of proteins^12^, stability at different pH values^13^ and oxidative environmental conditions^14^. These factors can disrupt stable folding and maturation of the chromophore or lead to rapid photobleaching.

High photochemical reactivity of cysteine residues is one of the reasons for these problems. Accordingly, amino acids such as alanine, valine, serine, threonine, and methionine were used as replacements for the amino acid residues of cysteine in several popular FPs (Table 1), generating MoxFP (“mox”-monomeric and oxidation resistant) proteins^14–17^.

**Table 1:** Amino acid substitutions in moxFPs.

FP	Ancestor FP	Amino acid substitution	References
*secBFP2*	mTagBFP	C29A/C118S	^17^
*moxBFP*	EBFP2	C48S/C70V/V163A/V206K	^14^
*moxDendra2*	Dendra2	N41Q/C101A/C113T/C171A/N200Q	^15^
*moxMaple3*	mMaple3	C110V/N111Q/C180A/N227D	^16^
*moxNeonGreen*	mNeonGreen	C139S	^14^
*moxCerulean3*	mCerulean3	C48S/C70S/N105T/S30R/Y39N/I171V	^14^
*moxGFP*	SuperfolderGFP	C48S/C70S/V206K	^14^
*cfSGFP2*	SGFP2	C48S/C70M	^18^

To date, there are three types of phototransformations: irreversible photoactivation, reversible photoswitching, and irreversible photoconversion. Photoactivatible fluorescent proteins (PAFPs) upon proper wavelength light irradiation irreversibly change from a non-fluorescent to a fluorescent state. Some of the representatives are PA-GFP^19^, PAmCherry^20^, PATagRFP^21^.

Photoconvertible fluorescent proteins (PCFPs) constitute a group of fluorescent proteins, starting with Kaede^22^ that, when exposed to proper light, bear the capability of irreversibly switching their emission color, generally from green to red. During this process, there is an irreversible break in the polypeptide chain, followed by rearrangement of conjugated bonds^23^ (Fig. S1).

Reversible switching fluorescent proteins (RSFPs) can repeatedly switch between fluorescent and non-fluorescent state under the influence of certain wavelengths of light. For most RSFPs, this occurs as a result of cis–trans isomerization of the chromophore^6,24,25^, except for protein Dreiklang^26^, it is characterized by the mechanism of covalent attachment and detachment of a water molecule close to the imdazolinone ring.

First well-studied RSFP was Dronpa^27^. It demonstrated the influence of key residues in the chromophore environment—157, 159, 173—on the properties of phototransformations^28–30^. Mutagenesis of these residues in the photoconvertible EosFP and Dendra proteins led to the production of biphotochromic variants: IrisFP^8^, NijiFP^9^, and Dendra-M159A^9^.Another biphotochromic protein pcDronpa was obtained by introduction C62H mutation into RSFP Dronpa^10^. SAASoti is a protein that is unique in structure because it is ancestorily biphotochromic, without requiring mutagenesis of the corresponding, or any other, residues to obtain these properties^31,32^ (Fig. S1, Fig. 2).

**Figure 1:**
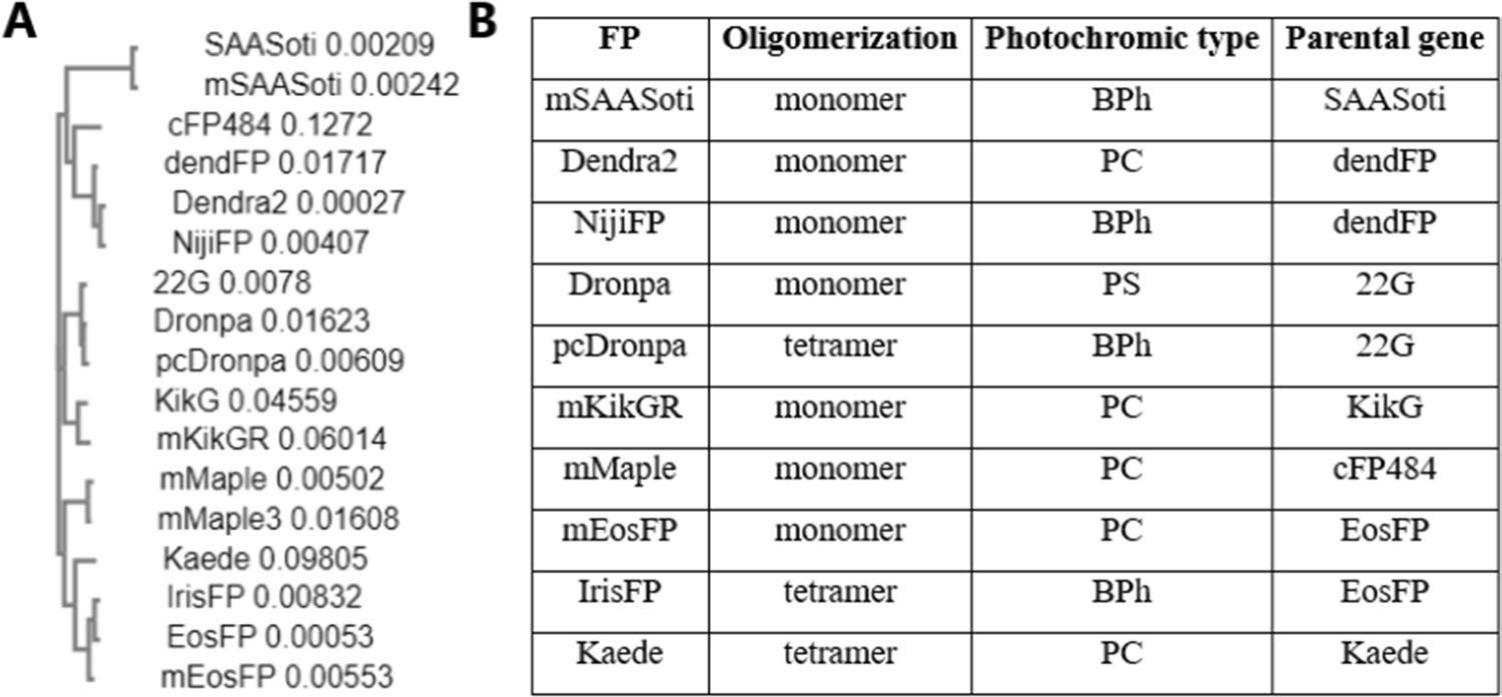
(**A**) Bayesian phylogenetic tree of the biphotochromic proteins and their ancestors. Numbers on the right represent branch lengths. (Data obtained with Clustal Omega tool https://www.ebi.ac.uk/Tools/services/rest/clustalo^33^). (**B**) Photochromic properties, oligomerization state and parental gene. *BPh* biphotochromic protein, *PC* photoconvertible protein, *PS* photoswitchable protein.

Phylogenetic analysis shows that, in terms of its primary structure, SAASoti is the most distant from similar phototransformable proteins, despite the fact that their sequences are more than 50% identical. (Fig. 1, Table S1).

In this work we set out to engineer a monomeric and cysteine-free variant of SAASoti (moxSAASoti). Notably, the replacement of each amino acid residue of cysteine led to unexpected effects, such as a shift in the pKa of the red form to the alkaline region, a change in the rate and depth of phototransformation^34^, and an attempt to combine all the substitutions turned out to be a nontrivial task.

## Results and discussion

As described previously^34^, the mSAASoti-3C (C21N/C71G/C175A mSAASoti mutant, mSAASoti is a SAASoti with V127T substitution (Table 2)) form was characterized by the fastest rates of green-to-red photoconversion and green-form photobleaching, and highest extinction coefficient (green form). The next aim was to develop the “mox” SAASoti form by substituting all 5 cysteine residues.

**Table 2:** Abbreviated names for the main SAASoti mutants discussed in the current work.

Parental variant	Substitutions	Name of new variant
SAASoti	V127T	mSAASoti
mSAASoti	C21N/C71G/C175A	mSAASoti-3C
mSAASoti-3C	C105G/C117V	moxSAASoti-V
mSAASoti-3C	C105G/C117T	moxSAASoti-T

The structural role of cysteine residues and the effect of their substitutions on the properties of fluorescent proteins have not yet been sufficiently studied. In some cases, mutation of cysteines to the other amino acid residues led to dim or dark proteins^15,17,35^.

**Figure 2:**
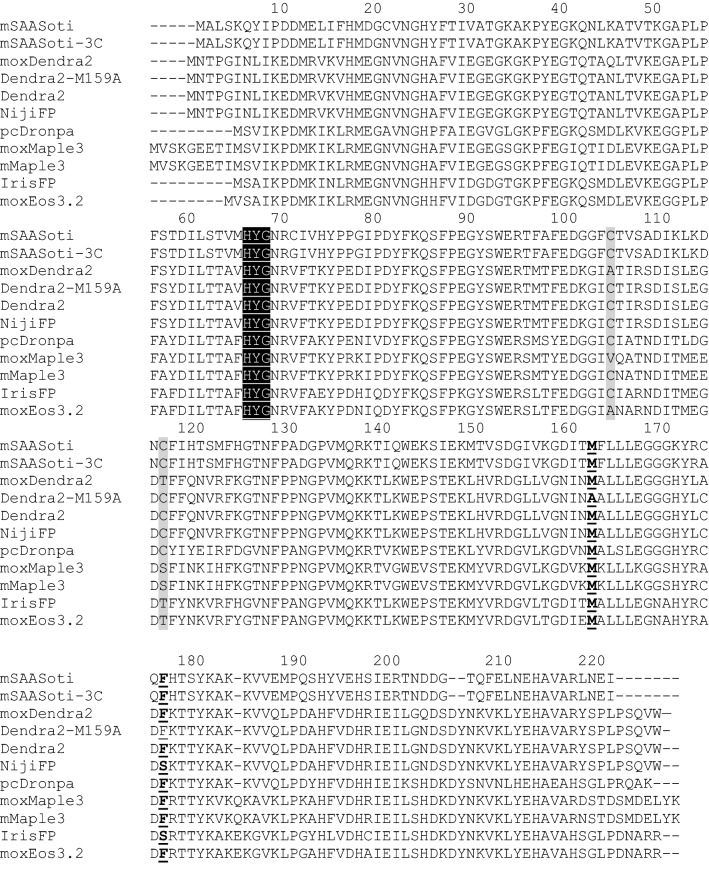
Sequence alignment of “mox” photoconvertible proteins, biphotochromic proteins and mSAASoti. The numbering is based on mSAASoti. Cysteine residues to mutate are highlighted in gray, chromophore is indicated by black shade, 163 and 177 residue is bold and underlined.

Based on aligned sequence with similar mox PCFPs and biphotochromic FPs (Fig. 2 and S2) we identified the most promising hotspots for cysteine replacement—residues 105 and 117.

mSAASoti-3C was used as a matrix. We applied site-saturated two-point mutagenesis for both substitutions simultaneously at both 105 and 117 residues, since the combination of single successful mutations led to the production of non-fluorescent proteins. According to the SAASoti 3D model (the 3D full atom model of the mSAASoti was previously obtained^34^ from the crystal structure of the IrisFP (PDB ID: 2VVH^8^)), residues at positions 105 and 117 are located on adjacent beta-sheets of the barrel. We hypothesized that combining two independently generated substitutions at these positions, acting alone, may disrupt beta-sheet interaction and disrupts the barrel structure, resulting in non-fluorescent protein variants.

The brightest clones contained glycine at position 105 and threonine or valine at position 117, New SAASoti mutants were expressed in *E. coli* cells and purified as described by a standard procedure^32^. The new variants were named moxSAASoti-V and moxSAASoti-T according to the sole alternative at position 117—valine or threonine.

Since the a.a. in position 117 is exposed on the surface in original mSAASoti-3C, its replacement could lead to increased aggregation of the new protein form. Therefore, the oligomerization state of new mutants was analyzed by size-exclusion chromatography. The elution volume (Table S2) and elution profiles (Fig. S3) for both mutants corresponded to a molecular weight of 25.7 kDa, which corresponded to a monomeric form of SAASoti. This findings may suggest that the external 117 residue does not play any important role in the oligomerization of moxSAASoti variants.

The most important thing was to analyze how substitutions influenced photochemical and photophysical properties. On-to-off green photoswitching kinetics are described by a biexponential model (Eq. (1)):

1 $$ I = I_{1} {*}\exp \left( { - k_{1} t} \right) + I_{2} {*}\exp \left( { - k_{2} t} \right) + c $$

The bi-exponential decay model during photoswitching is typical of most of the SAASoti variants published earlier^31,34^. A similar pattern was also observed for Dronpa ancestors^36^. Previously, it was hypothesized that the fluorescent proteins could exist in two different emissive states^37^; There are possible explanations for this phenomenon: (1) existence of several protein populations with different conformations of the protein environment of the chromophore^38^; (2) parallel photooxidation reaction proceeding with photoswitching, leading to the appearance of an oxidized fraction of a protein with a different photoswitching kinetics^34^.

**Figure 3:**
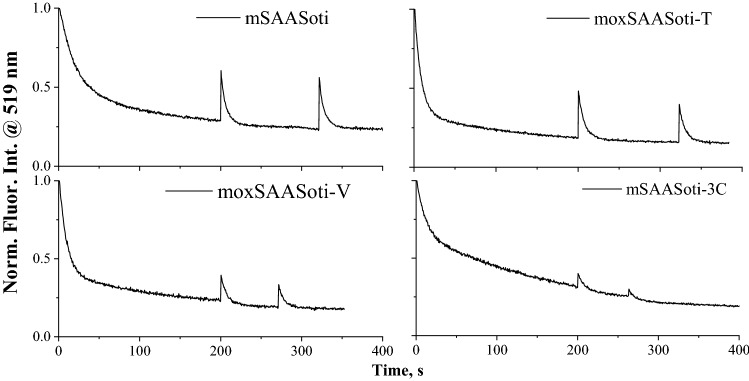
Normalized photoswitching profiles of mSAASoti variants. “Off” switching was induced by 470 nm illumination, “on” switching was induced by 400 nm illumination for 5 s. Proteins dissolved in 20 mM NaHCO_3_ buffer, pH 9.2.

On-to-off switching rates for new variants are very similar and larger than observed for mSAASoti-3C and parental mSAASoti: additionally, less photodestruction than for mSAASoti-3C was highlighted. (Fig. 3, Table 3).

**Table 3:** Kinetic parameters of “on-to-off”-switching, green to red photoconversion, calculated for mSAASoti mutants.

mSAASoti form	On-to-off photoswitching^a^			Green-to-red photoconversion^b^	
	k_1_ × 10^3^, s^−1^	k_2_ × 10^3^, s^−1^	I_1_/I_2_	k_1_ × 10^3^, s^−1^	k_2_ × 10^3^, s^−1^
mSAASoti	55.5 ± 1.0	8.3 ± 0.7	1.9	62 ± 1	55 ± 1
mSAASoti-3C	95.2 ± 2.0	5.0 ± 0.2	0.7	273 ± 16	106 ± 4
moxSAASoti-T	131.6 ± 0.9	7.5 ± 0.3	3.7	278 ± 16	79 ± 2
moxSAASoti-V	113.6 ± 1.3	7.3 ± 0.4	2.9	270 ± 14	107 ± 4

^a^Photoswitching kinetics is described by Eq. (1).

^b^Photoconversion kinetics is described by Eq. (2).

The moxSAASoti-V variant showed lower recovery after photoswitching than moxSAASoti-T (30% and 20% of the initial fluorescence intensity recovered after the first PS cycle in the case of moxSAASoti-T and moxSAASoti-V, respectively) and practically the same kinetic constants, indicating high rates of “on-to-off”-switching (Table 3 and Fig. 3). The fluorescence recovery after the second PS cycle is more complete, which probably indicates some photodestruction reaction in the first PS cycle. moxSAASoti-T shows the highest ratio of preexponential intensities, which indicates that the rapidly switching component (k_1_) makes the maximum contribution to the overall kinetics.

**Figure 4:**
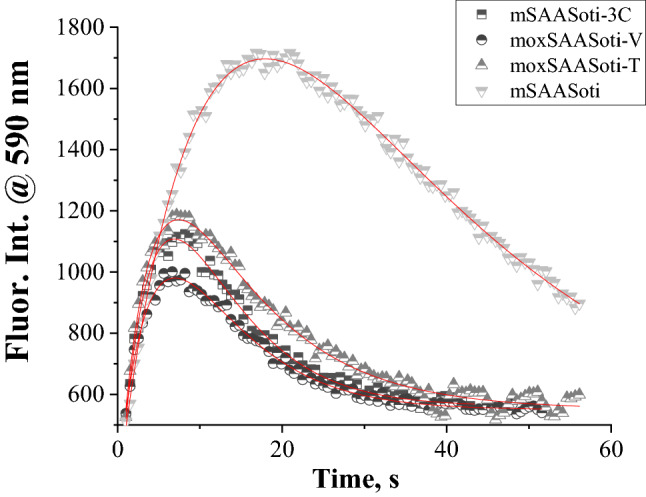
Green-to-red photoconversion of mSAASoti variants induced by 400 nm combined with 550 nm illumination. Experimental data are represented by dots, fitting by red lines. Protein samples prepared in 200 mM Tris–HCl, pH 7.4.

We tested the green to red photoconversion capacity of new variants. Mox variants such as their closest ancestor mSAASoti-3C showed a high green-to-red photoconversion rate but a low degree of photoconversion (Fig. 4). We assume several possible reasons for this fact: (1) low brightness of the red form and (2) unstable red form. It certainly could be a combination of both phenomena, which is the subject of further research.

The process of photoconversion was detected by changes in the red form fluorescence intensity (λ_em_ = 590 nm). It can be described by a bi-exponential model (Eq. (2)):

2 $$ I = - I_{1} {*}\exp \left( { - k_{1} t} \right) + I_{2} {*}\exp \left( { - k_{2} t} \right) + c $$

where the first exponent is responsible for the red form formation, while the second exponent describes its photodestruction, c—background and residual signal. The corresponding kinetic parameters (Table 3) for mox variants are close to the parent mSAASoti-3C, which could indicate that residues in positions 105 and 117 do not affect the green-to-red photoconversion.

We hypothesize that changes in the chromophore environment leading to an increase in the photoswitching rate, in turn, also impairs photoconversion. This may be due to the fact that the additional space in the chromophore environment, which facilitates cis–trans isomerization, hampers the stabilization of the red form of the chromophore, which decreases the efficiency of photoconversion. Currently, work is underway to confirm this hypothesis for SAASoti.

Based on the photochemical and photophysical properties of the new mox forms of the SAASoti protein, we chose the moxSAASoti-T variant for further characterization, since its on-to-off photoswitching rate is higher, off-to-on recovery is more complete and photoconversion rates are higher than those of mSAASoti.

We tested off-to-on thermal relaxation kinetics for moxSAASoti-T by observing the fluorescence at 520 nm. The relaxation kinetic constant value is 0.021 ± 0.001 min^−1^, which is up to 8 times greater than that for other variants with substituted cysteines and of the same order as mSAASoti, as previously published^34^.

We compared the main physicochemical properties (excitation/emission maxima λ_ex_/λ_em_, pK_a_ values of the chromophore, molar extinction coefficient (ε) and quantum yield (ϕ)) of moxSAASoti-T and other mSAASoti mutants with cysteine substitutions (Table 4). The excitation and emission maxima for the green and red forms did not change. Although the molar extinction coefficient decreased for the green form, however, it is still one of the highest among the cysteine variants of mSAASoti, and the reduced value of the extinction coefficient for the red form may be associated with a decrease in its stability. The fluorescence quantum yield of the green form also decreased by 0.1, and the molecular brightness of the new variant practically coincides with C117S and C21N/C175A mSAASoti. Interestingly, most impact new substitutions provided upon pK_a_ of the moxSAASoti red form. Previously, all cysteine substitutions resulted in higher pK_a_ values (except SAASoti C117S, which has the same mSAASoti pK_a_). We suggest that it could be an allosteric effect from T117 because a previous mutant with a single C117S substitution showed the lowest pK_a_ of the red form of all other mutants with single substitutions of cysteine residues.

**Table 4:** Physicochemical properties and fluorescent parameters of different mSAASoti mutant forms.

	λ_ex_/λ_em_	pK_a_	ε × 10^–3^, M^−1^ cm^-1^	ϕ	Brightness (ϕ × ε)	References
mSAASoti	509/519	6.3 ± 0.1	75.0	0.59 ± 0.02	44.3	^34^
	573/579	6.6 ± 0.1	24.0			
C21N	509/519	6.4 ± 0.1	82.4	0.61 ± 0.02	50.3	
	579/590	7.5 ± 0.1	25.4			
C105V	509/519	6.5 ± 0.1	61.0	0.60 ± 0.02	36.6	
	576/589	7.1 ± 0.1	16.4			
C71V	509/519	6.5 ± 0.1	65.1	0.63 ± 0.04	41.0	
	577/590	7.0 ± 0.1	12.3			
C175A	509/519	6.7 ± 0.1	80.1	0.55 ± 0.05	44.0	
	580/587	7.8 ± 0.1	22.9			
C117S	509/519	6.2 ± 0.1	66.3	0.54 ± 0.03	35.8	
	580/590	6.7 ± 0.1				
C21N/C71V	509/519	6.3 ± 0.1	48.9	0.58 ± 0.02	28.4	
	577/589	7.0 ± 0.1				
C21N/C175A	509/519	6.3 ± 0.1	65.4	0.55 ± 0.03	36.0	
	580/590	7.4 ± 0.1	12.7			
mSAASoti-3C	509/519	6.4 ± 0.1	83.8	0.60 ± 0.02	50.3	
	577/589	7.2 ± 0.2	14.8			
moxSAASoti	509/519	6.1 ± 0.1	71.8	0.50 ± 0.02	35.9	This work
	577/589	6.3 ± 0.1	11.3			

Point mutations in mSAASoti mainly affect the pK_a_ values (Table 4) of the red form and off-to-on switching rates of the green form (*k*_*off-to-on*_ for mSAASoti variants with cysteines substitutions, except moxSAASoti-T, were obtained in work^34^. Notably, single and triple mutations that are present in moxSAASoti-T considerably change these two macroscopic properties. However, substitutions of all cysteine residues result in the recovery of the mSAASoti properties. It is not evident which particular microscopic structural features are responsible for the changes in the macroscopic parameters. Previously^34^, it was demonstrated that the flexibility of F177 determines the rate of off-to-on photoswitching and that a change in the C–O distance of the phenyl fragment of the chromophore is responsible for the pK_a_ shift. Here, we test these notions on the set of five proteins of the mSAASoti family: the mSAASoti, its single mutants C21N and C175A and a triple mutant mSAASoti-3C with exactly the same point mutations as in the moxSAASoti, and moxSAASoti-T itself.

**Figure 5:**
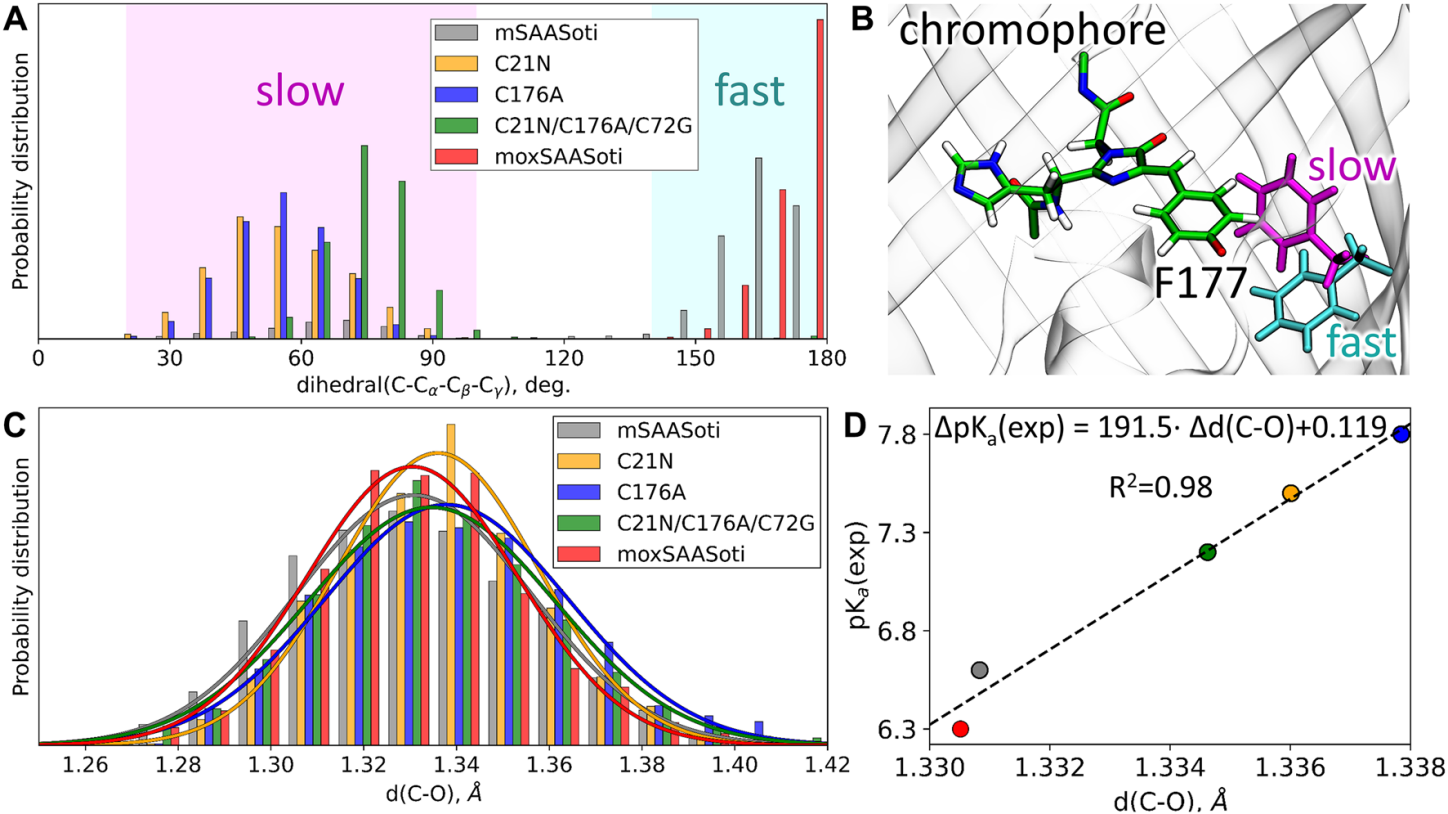
(**A**) Distributions of the C–C_α_–C_β_–C_γ_ dihedral of Phe177 in mSAASoti, its C21N, C175A and mSAASoti-3C mutants and moxSAASoti-T. (**B**) For the chromophore and two different conformations of the F177 residue, the conformation colored cyan corresponds to faster off-to-on isomerization and magenta corresponds to slower off-to-on isomerization. The images were drawn in program VMD 1.9.3 (https://www.ks.uiuc.edu/Research/vmd/vmd-1.9.3/). (**C**) Distributions of the C–O bond distances in the phenyl fragment of the neutral red form of the chromophore for different mSAASoti variants. (**D**) The relation between the calculated shifts of the mean values of d (C–O) and experimental pK_a_ shifts relative to the mSAASoti variant.

We performed a 200 ns classical MD run for each model system and analyzed the conformational behavior of the F177 side chain. As a measure of conformational diversity, we chose the dihedral angle C–C_α_–C_β_–C_γ_ (Fig. 5A,B). The mSAASoti and moxSAASoti-T variants predominantly demonstrate conformations with dihedral values between 140° and 180°. For slower proteins, single and triple mutants of mSAASoti, the dihedrals are distributed between 20° and 100°. This conformation is less favorable for off-to-on photoswitching, as the chromophore binding pocket is tighter in this case, which hinders isomerization (Fig. 5). Thus, the conformational flexibility of the F177 side chain could be the main responsible for the off-to-on photoswitching rate changes and can be further utilized as a measure of the photoswitching rate.

The C–O bond lengths in the substituted phenols are known to correlate with their pK_a_ values^39^. Here, we utilize this approach for more complicated systems, the same chromophore in slightly different protein environments due to point mutations. We perform QM/MM MD runs of the models comprising neutral red chromophores and analyze d (C–O) distributions (Fig. 5C). Although the chromophores in fluorescent proteins have a large number of interatomic interactions, the elongation of the C–O distance, which is a calculated parameter, reproduces the experimentally observed increase in the pK_a_ value (Fig. 5D).

This result is of great importance, as it demonstrates that even though we cannot distinguish the impact of each amino acid residue and its substitution, we can evaluate the d (C–O) values along the MD trajectories and use it as a calculated parameter to predict the pK_a_ value.

## Conclusions

In the present work, monomeric and cysteine-free moxSAASoti proteins were obtained. We hypothesize that these variants will have a more stable folding under the oxidative conditions of the cell.

MoxSAASoti was obtained from the triple mutant mSAASoti-3C by simultaneous random mutagenesis at positions 105 and 117, since, as we assume, due to the close location of these residues, working single substitutions disrupted the interaction of two adjacent β-sheets and destroyed the structure of the β-barrel. As a result of simultaneous random mutagenesis at two positions, two variants with bright fluorescence at 520 nm were obtained: moxSAASoti-T and moxSAASoti-V (containing substitutions of all cysteines, V127T and differing only in substitutions at position 117: C117T and C117V, respectively). Photoconversion and photoswitching properties were characterized for new variants in vitro in comparison with their ancestors, mSAASoti and mSAASoti-3C. The mox variants are characterized by a higher photoswitching rate compared to the mSAASoti and a decrease in the photobleaching rate compared to the mSAASoti-3C variant. The photoconversion rates are close to the mSAASoti variant and higher than that of mSAASoti-3C, however, the photoconversion efficiency is worse, due, as we assume, to a decrease in the stability of the red form. Since the kinetic parameters of moxSAASoti-V are slightly worse than those of moxSAASoti-T, the moxSAASoti-T variant was chosen as the basic moxSAASoti-T.

New substitutions mostly affect on pKa value of the red form. Using QM/MM MD simulations we find correlation between length of the d(C–O) bond of the phenyl fragment of the chromophore and pKa, Main importance is that it could possibly allow to predict the effect of a particular substitution on the value of pKa. Also, using classical MD simulations, we confirmed that the substitutions indirectly affect the mobility of 177 phenylalanine, which is responsible for the photoswitching rate. Since the altered residues are not located in close proximity to Phe177, we assume that they change the mobility of the beta sheets, freeing up and reducing the space for the movement of this residue.

Thus, we suggest that moxSAASoti-T can be used to study the dynamics of proteins in the oxidative and secretory environment, and its biphotochromic properties make it an interesting object for pulse-chase experiments in combination with PALM (photoactivation localization microscopy) or combination of pcSOFI and PALM.

## Materials and methods

### Mutagenesis and colonies screening

Site-saturated mutagenesis was performed by overlapping PCR with degenerate primers (Table 5). PCRs were carried out sequentially with each pair of primers using Pfu DNA polymerase. DNA with random substitutions in two positions (105 and 117) was cloned into the pEt22b vector and transformed into *E. coli* BL21(DE3) cells. The resulting colonies were transferred to LB agar medium with IPTG and grown at 20 °C for 24 h. Colonies were analyzed for fluorescence on an Olympus CKX41SF microscope by irradiation with excitation light at 470 nm.

**Table 5:** Degenerate primers for overlapped PCR.

Primer	Sequence
C105X_fw	GAT GGC GGA TTT NNN ACA GTC AGT GCA
C105X_rev	TGC ACT GAC TGT NNN AAA TCC GCC ATC
C117X_fw	GAC AAC NNN TTC ATT CAC ACA TCC ATG
C117X_rev	TGT GTG AAT GAA NNN GTT GTC TTT AAG TTT TAT

### Protein expression and purification

moxSAASoti was expressed and purified as described previously^32^, with the exception that cells were disrupted by ultrasonication. *Oligomerization analysis* by size-exclusion chromatography was performed as described earlier^32^. *Absorbance and fluorescence spectra* were detected using Cary 60 and Cary Eclipse, respectively, as described earlier^32^. *Green-to-red photoconversion and reversible photoswitching* experiments were performed using a homemade setup based on an Olympus CX41 upright microscope with Thorlabs (USA) light sources, 400 nm (and 560 nm for red form excitation) for photoconversion, and 470 nm for photoswitching using an Avesta ASP-75 spectrometer for detection. LED light was passed through Thorlabs MF390/18 and Chroma ET470/24 m bandpass filters. We obtained 282.4 and 706.1 mW/cm2 maximum power densities after Olympus 20x/0.4 Plan N objective. Protein solutions were placed in 5 μl glass capillaries. *Thermal relaxation* was analyzed by recording fluorescence intensities at 520 nm with 479 nm excitation. Intensities were recorded every 10 min to I = 1/2Imax values. *Data analyses* of all phototransformation experiments were performed with the OriginPro 2018 software package.

### Classical MD simulations

The 3D full atom model of the mSAASoti was previously obtained^34^ from the crystal structure of the IrisFP (PDB ID: 2VVH^8^). 3D models of C21N and C176A variants of the mSAASoti were also obtained in ref^34^. Here, we additionally prepared models of the triple mSAASoti-3C mutant and the mox form. The CHARMM36^40^ force field parameters were utilized for protein and the CGenFF^41^ force field parameters for the chromophore in the green form. The system was solvated in a rectangular water box with TIP3P^42^ water molecules and neutralized by adding sodium ions. Classical molecular dynamics simulations were performed in the NAMD software package^43^. Each system was preliminarily equilibrated by 10,000 minimization steps and a 20 ns MD run. Production runs for the mSAASoti and C21N variants of SAASoti were performed for 200 ns with a 1 fs time step in the NPT ensemble at p = 1 atm and T = 300 K. The pressure and temperature were controlled by a Nosé-Hoover barostat and Langevin thermostat, respectively. To decrease the influence of error accumulation for such long trajectories, we randomly reassigned velocities every 40 ns. The cutoff distances were 12 Å for both electrostatic and van der Waals interactions with switching to the smoothing function at 10 Å.

### MD simulations with QM/MM potentials

The systems for the QM/MM (combined quantum mechanics/molecular mechanics) MD simulations were preliminarily equilibrated in classical MD runs as described above. The simulations were performed for the same set of five systems, and the chromophore was in the neutral red form. The MM subsystems were described similarly to the classical MD. The QM part was composed of the chromophore, the side chains of Gln42, The63, Arg70, Arg95, Ser146, His197, Glu213 (in the neutral form) and two water molecules. The green to red conversion was manually performed, and these coordinates were used as initial for the QM/MM MD runs. The system was preliminarily minimized for 100 steps. After that the 5 ps production runs were performed. The QM part was described at the PBE0-D3/6-31G** Kohn–Sham DFT level^44,45^. The QM/MM MD simulations were performed using the interface^46^ for the classical MD software NAMD^43^ and the quantum chemistry package TeraChem^47^.

## Supplementary Information

Supplementary Information.
