# Supplementary material for: First biphotochromic fluorescent protein moxSAASoti stabilized for oxidizing environment
Source: Sci Rep. 2022 May 12;12:7862. doi: 10.1038/s41598-022-11249-x (PMC9098843; doi:10.1038/s41598-022-11249-x)
Supplement: Supplementary file 1 — Supplementary Information. [file 41598_2022_11249_MOESM1_ESM.docx]

**First biphotochromic fluorescent protein moxSAASoti stabilized**

**for oxidizing environment**

*Marynich N.K.^1^, Khrenova M.G.^1,2^, Gavshina A.V.^1^, Solovyev I.D.^1^, Savitsky A.P.^1, 2^*

^1^ A.N. Bach Institute of Biochemistry, Research Center of Biotechnology of the Russian Academy of Sciences, Moscow, Russia

^2^ Lomonosov Moscow State University, Department of Chemistry, Moscow, Russia

Supplementary

**Table S1.** Matrix of similarity of the green-to red photoconvertible proteins.

| 1 | SAASoti | 100 | 100 | 52 | 56 | 56 | 55 | 54 | 53 | 53 | 55 | 54 | 51 | 51 | 53 | 53 | 54 |
| --- | --- | --- | --- | --- | --- | --- | --- | --- | --- | --- | --- | --- | --- | --- | --- | --- | --- |
| 2 | mSAASoti | 100 | 100 | 52 | 56 | 56 | 56 | 54 | 53 | 53 | 55 | 55 | 52 | 50 | 53 | 53 | 53 |
| 3 | cFP484 | 52 | 52 | 100 | 77 | 74 | 74 | 69 | 67 | 67 | 65 | 64 | 72 | 65 | 68 | 69 | 69 |
| 4 | dendFP | 56 | 56 | 77 | 100 | 96 | 95 | 70 | 68 | 69 | 66 | 65 | 72 | 67 | 68 | 68 | 68 |
| 5 | Dendra2 | 56 | 56 | 74 | 96 | 100 | 100 | 69 | 67 | 68 | 65 | 64 | 70 | 67 | 67 | 68 | 68 |
| 6 | NijiFP | 55 | 56 | 74 | 95 | 100 | 100 | 68 | 67 | 67 | 64 | 64 | 70 | 66 | 68 | 68 | 68 |
| 7 | 22G | 54 | 54 | 69 | 70 | 69 | 68 | 100 | 97 | 97 | 83 | 76 | 77 | 74 | 77 | 77 | 78 |
| 8 | Dronpa | 53 | 53 | 67 | 68 | 67 | 67 | 97 | 100 | 98 | 81 | 75 | 75 | 72 | 75 | 75 | 75 |
| 9 | pcDronpa | 53 | 53 | 67 | 69 | 68 | 67 | 97 | 98 | 100 | 81 | 76 | 76 | 74 | 76 | 77 | 77 |
| 10 | KikG | 55 | 55 | 65 | 66 | 65 | 64 | 83 | 81 | 81 | 100 | 89 | 75 | 71 | 74 | 75 | 75 |
| 11 | mKikGR | 54 | 55 | 64 | 65 | 64 | 64 | 76 | 75 | 76 | 89 | 100 | 74 | 68 | 73 | 74 | 74 |
| 12 | mMaple | 51 | 52 | 72 | 72 | 70 | 70 | 77 | 75 | 76 | 75 | 74 | 100 | 74 | 75 | 77 | 76 |
| 13 | Kaede | 51 | 50 | 65 | 67 | 67 | 66 | 74 | 72 | 74 | 71 | 68 | 74 | 100 | 83 | 83 | 84 |
| 14 | IrisFP | 53 | 53 | 68 | 68 | 67 | 68 | 77 | 75 | 76 | 74 | 73 | 75 | 83 | 100 | 98 | 99 |
| 15 | mEosFP | 53 | 53 | 69 | 68 | 68 | 68 | 77 | 75 | 77 | 75 | 74 | 77 | 83 | 98 | 100 | 99 |
| 16 | EosFP | 54 | 53 | 69 | 68 | 68 | 68 | 78 | 75 | 77 | 75 | 74 | 76 | 84 | 99 | 99 | 100 |





**Figure S1.** Phototransformations of the SAASoti protein. A and B structures represent photoindused backbone cleavage during photoconversion. C and D structures - cis- and trans- isomers of the chromophore, formed under light illumination in the process of the reversible photoswitching

**Figure S2.** Alignment of the mSAASoti model (green) and Dendra2 (PDB ID: 2VZX, cyan). (A) The overall structure. (B) Residues of the chromophore binding pocket that might influence photoswitching. The images were drawn in program VMD 1.9.3 (https://www.ks.uiuc.edu/Research/vmd/vmd-1.9.3/)


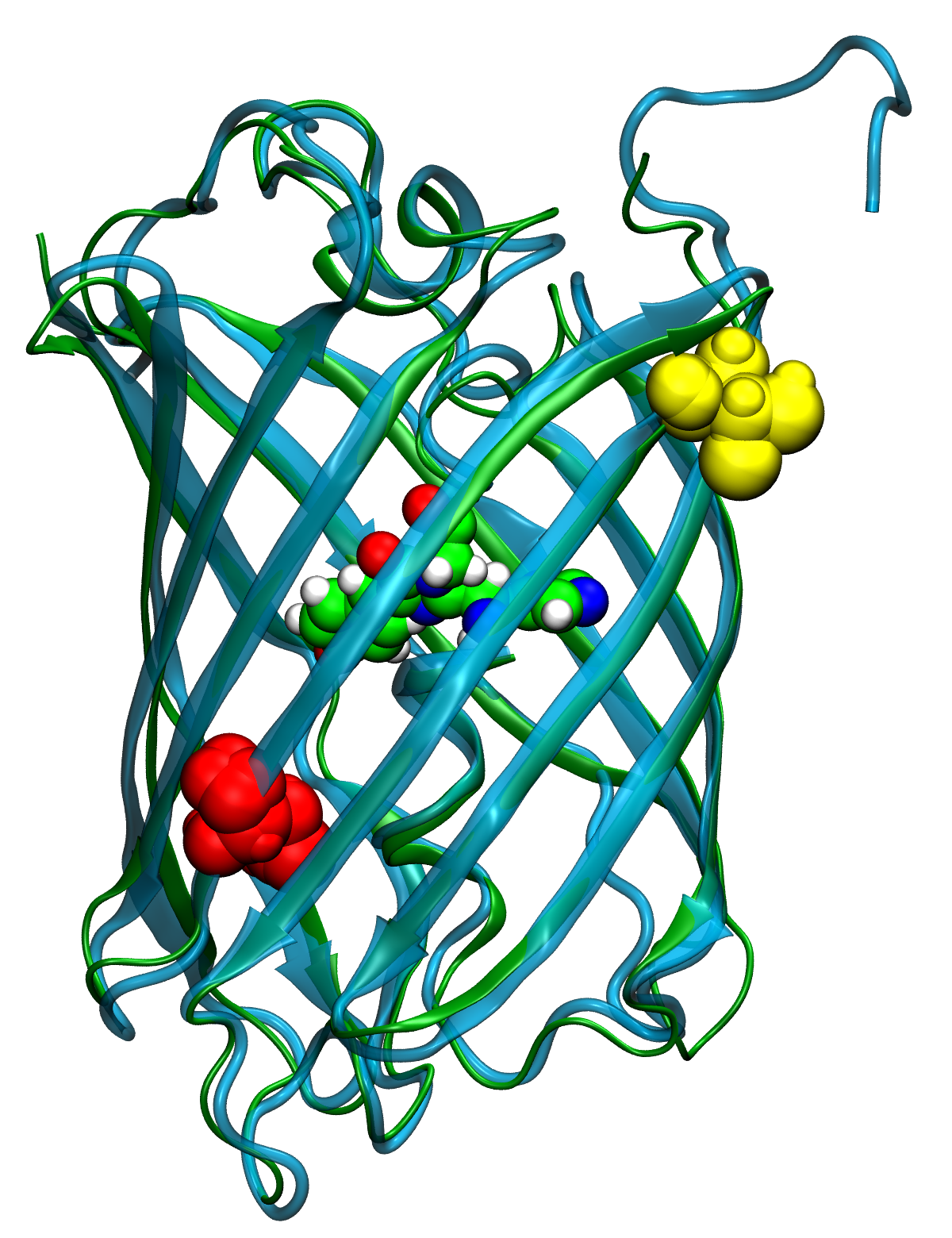


**Figure S3** 3D alignment of mSAASoti (cyan) and Dendra2 (green). Internal C105 residue colored in red, external C117 in yellow (numbering of residues correspond to SAASoti). The chromophore is colored by elements (carbon – green, nitrogen – blue, oxygen – red, hydrogen – white). The image was drawn in program VMD 1.9.3 (https://www.ks.uiuc.edu/Research/vmd/vmd-1.9.3/)

**Table S2.** Elution volume and calculated molecular weight of size-exclusion chromatography on Superdex200 column. Elution buffer 20 mM Tris-HC, 150 mM NaCl, pH 7.4

| “mox” variant | V, ml | Mw, kDa |
| --- | --- | --- |
| moxSAASoti-T | 16.8 | 25.7 |
| moxSAASoti-V | 16.8 | 25.7 |

**Figure S4.** Elution profiles of size-exclusion chromatography for two “mox” variants of SAASoti protein. Superdex 200 100/20 GL column, detection by absorption at 509 nm, 20 mM Tris-HCl, 150 mM NaCl buffer (pH 7.4).
